# Immune Microenvironment in Osteosarcoma: Components, Therapeutic Strategies and Clinical Applications

**DOI:** 10.3389/fimmu.2022.907550

**Published:** 2022-06-01

**Authors:** Tianyi Zhu, Jing Han, Liu Yang, Zhengdong Cai, Wei Sun, Yingqi Hua, Jing Xu

**Affiliations:** Department of Orthopedics, Shanghai General Hospital, Shanghai Jiao Tong University School of Medicine, Shanghai Bone Tumor Institution, Shanghai, China

**Keywords:** osteosarcoma, immune microenvironment, therapeutic strategies, clinical applications, immune cells, non-immune cells, complement, exosomes

## Abstract

Osteosarcoma is a primary malignant tumor that tends to threaten children and adolescents, and the 5-year event-free survival rate has not improved significantly in the past three decades, bringing grief and economic burden to patients and society. To date, the genetic background and oncogenesis mechanisms of osteosarcoma remain unclear, impeding further research. The tumor immune microenvironment has become a recent research hot spot, providing novel but valuable insight into tumor heterogeneity and multifaceted mechanisms of tumor progression and metastasis. However, the immune microenvironment in osteosarcoma has been vigorously discussed, and the landscape of immune and non-immune component infiltration has been intensively investigated. Here, we summarize the current knowledge of the classification, features, and functions of the main infiltrating cells, complement system, and exosomes in the osteosarcoma immune microenvironment. In each section, we also highlight the complex crosstalk network among them and the corresponding potential therapeutic strategies and clinical applications to deepen our understanding of osteosarcoma and provide a reference for imminent effective therapies with reduced adverse effects.

## 1 Introduction

Osteosarcoma is a rare primary cancer, characterized by the production of an abnormal and immature osteoid matrix (1). Despite its rarity in the whole spectrum of diseases, with an annual incidence rate of 4.7 per million, osteosarcoma ranks first among malignant bone tumors in young people (0–19 years) and has complex heterogeneity (2). In certain circumstances, osteosarcoma is associated with or secondary to other diseases, such as Paget’s disease, retinoblastoma, Li–Fraumeni syndrome, Rothmund–Thomson syndrome, and Bloom syndrome, which may be rooted in genetic risks, adding to the complexity of the condition (3, 4). The primary clinical manifestations of osteosarcoma are bone pain, swelling, and functional impairment. As the onset is usually insidious, it may not be taken seriously in the early stages. Another terrible situation is misdiagnosis as osteomyelitis, benign tumors, or metastatic bone tumors, which consequently leads to improper treatment (5). Osteosarcoma treatment is based on its classification and staging. A combination of surgery and chemotherapy is the first choice of treatment for high-grade osteosarcoma. Chemotherapy is considered to be applied preoperatively or postoperatively, according to specific conditions. For low-grade osteosarcoma, surgery alone is no worse than surgery plus chemotherapy (6). Surgery is also the preferred option for resectable metastases and pathological fractures (5, 7). The MAP regimen, comprising doxorubicin, cisplatin, and high-dose methotrexate, is the cornerstone of chemotherapy. It is worth noting that the impact of methotrexate on older adult patients is unpredictable and lacks positive evidence. Therefore, replacing methotrexate with ifosfamide is recommended for patients over 40 years of age. Second-line chemotherapy includes ifosfamide, cyclophosphamide, etoposide, carboplatin, gemcitabine, docetaxel, sorafenib, regorafenib, and samarium (8–15). Muramyl tripeptide is an innate immunomodulatory drug that has already been approved in Europe for the treatment of patients under the age of 30 years with resected osteosarcoma (16). Despite such exploration, the 5-year event-free survival rate of 70% for patients with osteosarcoma has not improved significantly over the last three decades, which indicates that existing regimens remain insufficient and limited. There is great variation among different individuals in response to the same regimen of therapeutic management. Therefore, there is still a long way to go for osteosarcoma treatment research.

Currently, the focus on tumors has expanded from the tumor cell itself to the tumor environment, in which tumor cells are promoted to uncontrollably proliferate, migrate, and resist apoptosis and drugs. An increasing number of studies have shown that changes in the tumor microenvironment are important (17). The immune microenvironment is a novel perspective to view and interpret, and its overall feature is immune suppression to help tumor cells escape immune surveillance. Components of the immune microenvironment of osteosarcoma are mainly divided into two categories: cellular and acellular substances. The former includes immunocytes, such as tumor-associated macrophages (TAMs), tumor-associated neutrophils (TANs), myeloid-derived suppressor cells (MDSCs), mast cells (MCs), T cells, B cells, natural killer cells (NK cells), and dendritic cells (DCs). Non-immune cells, including mesenchymal stem cells (MSCs) and circulating tumor cells (CTCs), can actively interact with the immune system and promote the formation of inhibitory immune networks (18). The complement system and exosomes with special immune effects are also hot spots in the field of microenvironment research.

Surgery and stereotactic radiotherapy can largely remove localized tumors at early stages. However, both approaches are limited by space and cannot eradicate all osteosarcoma cells in the body, especially metastatic and circulating osteosarcoma cells, which may lead to relapse and progression (19, 20). Studies have shown that tumor cells may be in constant confrontation with the immune system, and the balance can be disrupted at a certain time point. Once tumors are generated, they are difficult to completely remove. Immunity is promising for eliminating tumor cells from the body at the cellular level (21). Drugs that target the immune microenvironment are gradually stepping onto the stage with great application potential.

**Figure 1:**
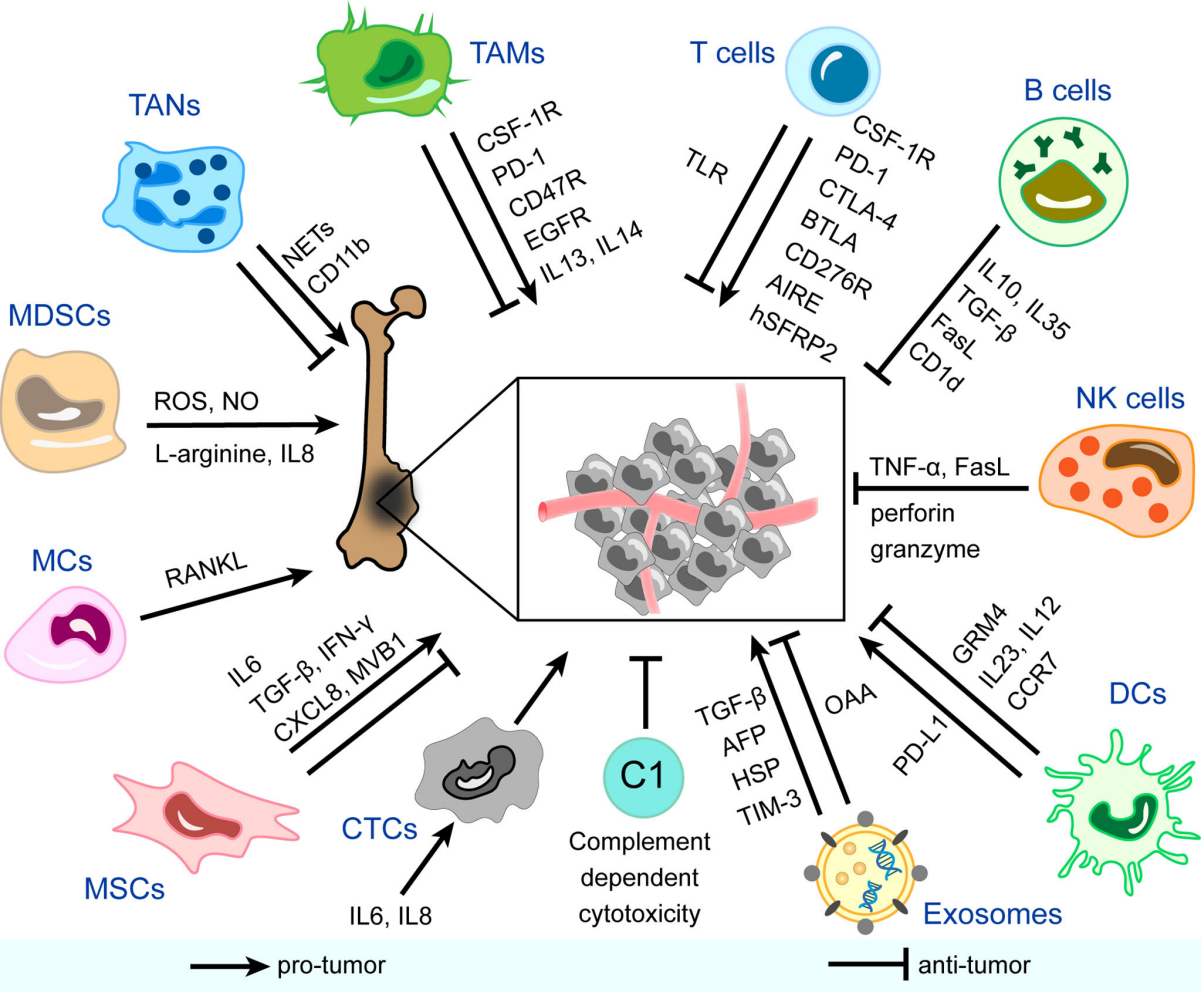
Immune and non-immune components in the immune microenvironment of osteosarcoma and mechanisms of their pro-tumor/anti-tumor effects. CSF-1R, colony-stimulating factor 1 receptor; PD-1, programmed cell death protein-1; EGFR, epidermal growth factor receptor; IL, interleukin; NETs, neutrophil extracellular traps; ROS, reactive oxygen species; NO, nitric oxide; RANKL, receptor activator NF-κB ligand; TGF-β, transforming growth factor-beta; IFN-γ, interferon-gamma; CXCL8, C-X-C motif chemokine ligand 8; AFP, α-fetoprotein; HSP, heat shock protein; TIM-3, T cell immunoglobulin and mucin domain-containing protein-3; OAA, osteosarcoma-associated antigens; PD-L1, programmed cell death protein ligand-1; GRM4, glutamate metabotropic receptor 4; CCR7, chemokine receptor 7; TNF-α, tumor necrosis factor-alpha; CTLA-4, cytotoxic T-lymphocyte-associated protein-4; BTLA, B And T-lymphocyte attenuator; AIRE, autoimmune regulator expression; hSFRP2, humanized secreted frizzled-related protein 2; TLR, toll-like receptor; TAMs, tumor-associated macrophages; TANs, tumor-associated neutrophils; MDSCs, myeloid-derived suppressor cells; MCs, mast cells; MSCs, mesenchymal stem cells; CTCs, circulating tumor cells; C, complement; DCs, dendritic cells; NK cells, natural killer cells.

Few reviews have focused on the panorama of the immune microenvironment in osteosarcoma specially. Instead of rigidly borrowing conclusions from other studies on the immune microenvironment in other solid tumors, this review systematically summarizes the main components in the immune microenvironment of osteosarcoma and their functional characteristics, as is shown in **Figure 1**. In each column, we also list relevant therapeutic strategies and clinical applications in progress. The ultimate aim is to provide more information and insight into the understanding and treatment of osteosarcoma.

## 2 Cells

### 2.1 Myeloid Cells

#### 2.1.1 Tumor-Associated Macrophages

TAMs are the most abundant tumor cells in the immune microenvironment of osteosarcoma and account for approximately 50% of the total tumor volume (22). TAMs play important roles in matrix remodeling, inflammation, vascularization, immune defense, and regulation. There are two TAM phenotypes: M1 which is classically activated and M2 which is alternatively activated. Generally, the M1 phenotype participates in inflammation with a reversed impact on metastasis, whereas the M2 phenotype is involved in wound healing and immune regulation with an accelerative impact on metastasis (23). The exact mechanisms of the complex impact include gene alteration, Notch pathway abnormality, macrophage polarization, and helper T cells (Th) 1/Th2 cytokine disturbance (24–26). Therefore, as the understanding of TAMs has deepened, researchers have attempted to inhibit M2 polarization in various ways to prevent tumor progression.

##### Therapeutic Strategies

All-trans retinoic acid prevents the migration of osteosarcoma cells both *in vitro* and *in vivo* by inhibiting interleukin (IL) 13- or IL14-induced M2 polarization (23). Another study indicated that all-trans retinoic acid could decrease cancer stem cell (CSC) properties strengthened by the M2 phenotype, increasing the number of CD117^+^Stro-1^+^ cells and the overexpression of CD133, CXCR4, Nanog, and Oct4 (27). Therefore, all-trans retinoic acid is expected to be added to the existing standard regimens. Paradoxically, studies have suggested that the M2 phenotype might be anti-tumor and anti-metastatic in osteosarcoma (24, 28). Classifying TAMs into M1 and M2 subgroups to analyze their influence on osteosarcoma pathogenesis, metastasis, and drug resistance may be crude and one-sided. Researchers are also attempting other approaches targeting TAMs to treat osteosarcoma. In human osteosarcoma implantation mice, scientists utilized a specific macrophage-eliminating liposome to ablate TAMs, which led to decreased tumor growth. Moreover, when epidermal growth factor receptor was silenced by short hairpin RNA in implanted osteosarcoma cells, tumor growth stimulated by recruited and polarized macrophages was suppressed (29). TAM-specific surface molecules are ideal targets for drug development. CD47R and programmed cell death protein-1 (PD-1) are two popular surface molecules that can be used to generate immune checkpoint inhibitors. Mifamotide and camrelizumab are being tested for their pharmacological effects on blood and lymphatic vessel formation, immunosuppression, and drug resistance (22). Pexidartinib (PLX3397), an inhibitor of colony-stimulating factor 1 receptor, has the potential to reprogram TAMs and activate T cells infiltration in osteosarcoma, resulting in decreased tumor growth and lung metastasis (30). In summary, TAMs are potential target candidates for new therapies.

#### 2.1.2 Tumor-Associated Neutrophils

Most research on neutrophils in patients with osteosarcoma focuses on the neutrophil-lymphocyte ratio or circulating neutrophils. Increased pre-treatment or preoperative neutrophil-lymphocyte ratio might be correlated with poor outcomes, which means that neutrophil-lymphocyte ratio can be investigated as a prognostic biomarker (31–35). However, there has been insufficient research on neutrophil infiltration in osteosarcoma microenvironment. Neutrophils in the tumor immune environment, known as TANs, exhibit phenotypic heterogeneity and functional versatility (36). In osteosarcoma, research on TANs is still in its early stages. The lifespan of TANs may be longer than that of circulating neutrophils under stimulation by pro-inflammatory factors such as interferon-gamma (IFN-γ) (37, 38). Neutrophil extracellular traps are web-like chromatin structures formed by granule proteins and chromatin secreted by neutrophils. Unlike traditional phagocytosis and killing factor secretion, neutrophil extracellular traps can promote metastasis *via* the DNA receptor coiled-coil domain containing protein 25 (39). Leshner et al. (40) also found that peptidylarginine deiminase 4, which is vital for extensive chromatin decondensation to form neutrophil extracellular traps, is overexpressed in osteosarcoma. Similar to the M1 and M2 subtypes of TAMs, Fridlender et al. (41) reported that TANs could also be polarized to the anti-tumor N1 phenotype and pro-tumor N2 phenotype.

Yang et al. (42) found that the number of neutrophils in non-metastatic tissues (detected by the neutrophil-specific marker CD11b) was higher than that in metastatic tissues. The infiltrated neutrophils exerted anti-tumor effects by coordinating the recruitment of immune cells, but also mediating antibody−dependent cellular cytotoxicity. In addition, neutrophil infiltration has been suggested to correlate with hypoxia-associated genes. Emerging evidence indicates that the hypoxic microenvironment plays a pivotal role in tumor progression, and a retrospective study assessed the validation of hypoxia-associated risk score as a prognostic factor of metastasis. The results of this study indicated that TANs were downregulated in the high hypoxia-risk group. The authors concluded that hypoxia might downregulate anti-tumor immune cells, which contribute to immune escape and metastasis (43). Unfortunately, both studies mentioned above did not take functional differences between subtypes into consideration, but merely counted the total number of TANs. This may be because of the difficulty in identifying the ideal markers. More detailed research is needed to uncover the complex role of TANs in the immune microenvironment of osteosarcoma. Development of therapeutic strategies associated with TANs in osteosarcoma is still ongoing and require creative ideas based on basic scientific research.

#### 2.1.3 Myeloid-Derived Suppressor Cells

MDSCs are a population of heterogeneous immunosuppressive immature myeloid cells that can differentiate into TAMs, TANs, and tumor-associated DCs. MDSCs not only interact with immune substances but also closely interact with osteoclasts, osteoblasts, chondrocytes, and other stromal cells in the bone and joint microenvironment to promote the pathogenesis and metastasis of osteosarcoma. MDSCs are classified as granulocytic MDSCs/polymorphonuclear MDSCs (G-MDSCs/PMN-MDSCs) and monocytic MDSCs (M-MDSCs). Recent studies have identified early bone marrow mesenchymal stem cells (e-MDSCs) that act as precursors of both PMN-MDSCs and M-MDSCs (44).

Among all immune cells, MDSCs interact with T cells most closely, which exerts the effect of inhibiting proliferation of T cells, reducing T cell-mediated immune responses and promoting T cell apoptosis by consuming L-arginine and producing reactive oxygen species in the microenvironment. Different MDSC subpopulations undergo different pathways to inhibit T cell function. PMN-MDSCs produce reactive oxygen species mainly by activating signal transducer and activator of transcription (STAT) 3 and upregulating nicotinamide adenine dinucleotide phosphate oxidase, whereas M-MDSCs produce nitric oxide mainly by activating STAT1 and upregulating inducible nitric oxide synthase to inhibit the effect of T cells. MDSCs suppress not only acquired anti-tumor immunity but also innate anti-tumor immunity. In addition to T cells, MDSCs inhibit the function of NK cells and DCs (45–47). Interestingly, stimulated by the hypoxic microenvironment, MDSCs express high levels of vascular endothelial growth factor, vascular endothelial growth factor analog Bv8, basic fibroblast growth factor, and matrix metalloprotease 9 to facilitate angiogenesis and the formation of a pre-metastatic niche, which has a strong relationship with osteosarcoma metastasis (48).

##### Therapeutic Strategies and Clinical Applications

Because MDSCs extensively infiltrate osteosarcoma lesions and inhibit anti-tumor immunity, researchers have been inspired to develop related therapies. The process of obliterating osteosarcoma cells with some existing drugs involves modulation of MDSCs immune responses. Studies have shown that current neoadjuvant chemotherapeutic drugs (doxorubicin, cisplatin, ifosfamide) could reduce the number of MDSCs in osteosarcoma patients, boost local immune states, and increase immune sensitivity (49). All-trans retinoic acid has been found to affect not only TAMs but also MDSCs by reducing the number of M-MDSCs and the potency of PMN-MDSCs (50, 51). Metformin has been shown to modulate the metabolism of MDSCs to play an anti-tumor role in osteosarcoma by downregulating oxidative phosphorylation and upregulating glycolysis, which is also related to the enhancement of T cell immunity (52). MDSCs can also be targets of the drugs themselves. Tumor cell surface vimentin-targeted interleukin 12 alters the immune profile (IFN-γ^Hi^CD8^Hi^FOXP3^Low^CD33^Low^) in mice transplanted with osteosarcoma and lowers the number of MDSCs, thereby controlling tumor recurrence and metastasis (53). Because infiltrating MDSCs in the osteosarcoma microenvironment express the chemokine receptor CXCR4, Jiang et al. (54) designed an antagonist of CXCR4, AMD3100, and tested its synergistic effect in combination with an anti-PD-1 antibody in an osteosarcoma murine model. In addition, Shi et al. (55) combined a functional inhibitor of PMN-MDSCs *via* selectively suppressing PI3Kδ/γ, (S)- (–)-N-[2-(3-Hydroxy-1H-indol-3-yl)-methyl]-acetamide (SNA), with an anti-PD-1 antibody to treat mice bearing osteosarcoma, and they validated that tumor growth was restrained and survival time was prolonged. Other studies have attempted to inhibit osteosarcoma progression by preventing the migration of MDSCs to the tumor microenvironment. Guan et al. (56) found that in an osteosarcoma murine model, anti-IL18 therapy significantly reduced the abnormal upregulation of MDSCs in peripheral blood, thus effectively curbing chemotaxis and infiltration, and finally inhibiting tumor progression. In addition to serving as a drug target, the number of MDSCs in the peripheral blood or tumor microenvironment of osteosarcoma is also a promising candidate as a prognostic biomarker (57). However, owing to the lack of highly specific markers for MDSCs, MDSCs-related therapy of osteosarcoma has not been sufficiently safe, and further research on its identification markers is needed. The mechanisms of accumulation, migration, and interaction with other immune and non-immune cells of MDSCs are also a mystery and require more effort.

#### 2.1.4 Mast Cells

MCs rank among the top five infiltrating cells in osteosarcoma and can be classified into resting MCs and activated MCs. The level of activated MCs in the osteosarcoma microenvironment is significantly higher than that in the normal set (58–60). The infiltration of MCs (CD117^+^ and tryptase^+^) was lower in the center of the tumor mass and more distributed at the normal-tumor interface where osteolysis occurs. The special distribution of MCs may be related to their function. Heymann et al. (61) found that under the influence of osteosarcoma cells, MCs could maintain viability and activity and produce receptor activator NF-κB ligand, a key molecular triad controlling bone remodeling. The dissolution and reconstruction of bone can help immunosuppressive cells further infiltrate the tumor microenvironment and shield the immune escape of tumor cells. Therefore, Inagaki et al. (62) proposed that MCs could function as biomarkers for osteolysis.

##### Clinical Applications

The most popular application of MCs in osteosarcoma is as a prognostic marker. MCs have been found to have the potential to predict metastasis and survival. Fan et al. (63) reported that the abundance of activated MCs in osteosarcoma microenvironment is associated with negative outcomes, which might indicate the prognosis of patients. Wei et al. (64) detected a correlation between immune-related genes and long noncoding RNAs to compare the different landscapes of immune-related long noncoding RNA pairs in localized and metastatic osteosarcoma. A significant difference in immune infiltration was observed between localized and metastatic osteosarcoma, and the high abundance of activated MCs indicated unsatisfactory outcomes. Le et al. (58) found that the proportion of MCs in patients who died was higher than that in living patients, implying a negative association between MCs and prognosis. In general, because of the unclear role of MCs in the immune microenvironment, related therapies are just beginning and urgently require further development.

#### 2.1.5 Dendritic Cells

DCs are derived from the bone marrow and can be divided into three major subgroups: plasmacytoid DC, myeloid/conventional DC1, and myeloid/conventional DC2. DCs act as a bridge between innate and adaptive immunity and are the most important antigen-presenting cells (65). Inflammatory infiltration varies markedly among different types of sarcomas, and DCs do not differ. There are more infiltrating cells represented by (DC-SIGN/CD11c^+^) DCs, CD14^+^/CD68^+^ TAMs, and CD3^+^ T cells in conventional high-grade osteosarcoma, undifferentiated pleomorphic sarcoma, and giant cell tumor of the bone than in Ewing’s sarcoma, chordoma, and chondrosarcoma (62). Another study found that the quantity of resting DCs was significantly higher in tissues with high immune scores in contrast to the low immune score group, and the degree of DC activation was positively correlated with outcomes (60). Furthermore, the infiltration of DCs into osteosarcoma tissues was found to be related to autophagy. Zhang et al. (66) tested the correlation between immune cell infiltration and 13 autophagy-related long noncoding RNAs, one of which was named RUSC1-AS1 and was negatively associated with the proportion of infiltrating immature DCs, macrophages, and mast cells. The study also illustrated that the level of plasmacytoid DCs was higher in the osteosarcoma microenvironment of the high-risk group than in the low-risk group, whereas the levels of total DCs and immature DCs were lower and associated with poor prognosis. However, the subgroup and quantity of DCs in the tumor microenvironment of the same patient are not static but dynamic in the trend of first increasing and then decreasing in quantity. DCs can trigger further immune responses by detecting tumor antigens and presenting them to helper and cytotoxic T cells, during which time they transform from immature DCs to mature DCs. Therefore, in the early stages, DCs proliferate actively and mature to activate helper and cytotoxic T cells. As the tumor grows, osteosarcoma cells develop variants resistant to DCs and phagocytes, leading to less stimulation of DC activation and eventual immune escape (67).

DCs are known to drive the pathogenesis of osteosarcoma through oncogenes and the tumor suppressor glutamate metabotropic receptor 4. Glutamate metabotropic receptor 4^-^/^-^ DCs secrete more IL23 and IL12 than wild-type DCs, leading to rapid tumor growth and accelerated progression in mouse models. DCs cultivated with osteosarcoma cells express increased IL23 and decreased IL12, and the higher ratio of IL23/IL12 can be reduced by augmented glutamate metabotropic receptor 4 signaling. Agonists of glutamate metabotropic receptor 4 or an antibody against IL23 may be promising treatment candidates (68, 69). DCs may also be associated with metastasis in patients with advanced osteosarcoma. A study on the single-cell RNA landscape revealed that CCR7 participates in the deformation, chemotaxis, migration, and survival of DCs, which are crucial to tumor metastasis. The study also demonstrated that compared with primary and recurrent lesions, the proportion of CD1c^+^ DCs is large in lung metastatic lesions (70). Although several lines of evidence have drawn a beneficial portrait of DCs in osteosarcoma, some studies have reported contradictory results. Koirala et al. (71) examined the role of PD-L1 and explored its prognostic value. They concluded that PD-L1 is significantly associated with DCs, T cells, and NK cells. Furthermore, DCs (28.3% vs. 83.9%, P=0.001) and TAMs (45.5% vs. 84.4%, P=0.032) were significantly associated with worse 5-year event-free survival. Another study investigating the immune classification in osteosarcoma also showed a negative association between DCs and prognosis. Their analysis suggested that the number of DCs in live patients was less than that in dead patients, in contrast to NK cells and CD8^+^ T cells (58).

##### Therapeutic Strategies

Scientific research has shed light on the therapeutic potential of DCs, and scientists have achieved some inspiring success. Some agents or partial components of the agents enhance the impact of DCs. For example, capsaicin was reported to enhance the phagocytosis of osteosarcoma cells (MG-63) by DCs *in vitro (* 72). The most popular treatment approach for DCs is vaccination. Several vaccines have shown encouraging efficacy, such as the CD1c^+^ DC vaccine and vaccination with polyinosinic:polycytidylic acid (poly I:C) activated and tumor antigen-loaded CD103^+^ myeloid/conventional DC1s (70, 73). In addition to vaccines, liposomal-muramyl tripeptide phosphatidylethanolamine has a good chance of extending overall survival and survival without metastasis by charging DCs or producing T cells, not only when used alone but also in combination with other approaches (74). Scientists have already investigated the effect of DCs to explore the possibility of their application in combination with anti-transforming growth factor-β (TGF-β) antibodies, agonist anti-glucocorticoid-induced tumor necrosis factor receptor antibodies, and doxorubicin (60, 67, 75). For example, Kawano et al. (76) combined DCs and anti-TGF-β antibodies to treat osteosarcoma and detected enhanced systematic immune responses *in vivo*. Existing attempts to utilize DCs to maximize tumor killing by virtue of upregulating the immunocompetence of lymphocytes. Nevertheless, the studies mentioned above also focused on the pro-tumor activity of DCs, which is a vital risk when using DC-associated therapy. Making full use of the advantages and avoiding the disadvantages with further precision therapy is the key to DCs application in the future.

### 2.2 Lymphoid Cells

#### 2.2.1 T Cells

T cells are thymus-derived lymphocytes that mature and reside in thymus-dependent areas of peripheral immune organs. T cells play a vital role in both cellular and humoral immunities. The classification of T cells according to different criteria is very complex. In the activation stage, T cells can be divided into naive, effector, and memory T cells. According to the T cell receptor characteristics, including distribution and major histocompatibility complex restriction, T cells can be divided into αβT and γδT. On the principle of function, T cells can be divided into Th, including Th1, Th2, Th9, Th17, Th22, and follicular helper T cells, cytotoxic T lymphocytes (CTLs), and regulatory T cells (Tregs), including natural Tregs, inducible Tregs, and other Tregs.

T cell infiltration plays a critical role in osteosarcoma anti-tumor immunity, and its classification is highly heterogeneous. In osteosarcoma, tumor-infiltrating lymphocytes are mainly distributed in the region expressing human leukocyte antigen Class I, whereas CD4^+^ and CD8^+^ T cells are mainly clustered at the interface between pulmonary metastases and normal tissues (57). The number of T cells in metastatic lesions is significantly higher than that in primary and recurrent lesions *in situ *(77). In metastatic lesions, checkpoint and immunoregulatory molecules were calculated to be higher than those in primary lesions, including PD-1, lymphocyte activation gene-3, T cell immunoglobulin and mucin domain-containing protein-3 (TIM-3), indoleamine 2,3-dioxygenase 1, and IFN-γ (57). Han et al. (78) analyzed the biopsy tissue and peripheral blood of 16 patients with primary osteosarcoma and concluded that there were more TIM-3^+^PD-1^−^ T and TIM-3^+^PD-1^+^ T cells in biopsy tissue than in peripheral blood, suggesting that the immune microenvironment in tumor lesions was inhibitory. They also reported that immune cells could interact with each other to form a vicious cycle, in which the immune activity of T cells could be inhibited by pro-tumor macrophages, and depletion of CD163^+^ macrophages could increase T cell growth and pro-inflammatory factor production *in vitro*. Another interesting case report from Japanese Hiroshima University was the case of extraosseous osteosarcoma with partial spontaneous regression and CD8^+^T cells, T cell-restricted intracellular antigen-1^+^ T cells, and granzyme B^+^ T cells in the tumor mass (79). These studies suggest that sophisticated T cell infiltration occurs in osteosarcoma in terms of regions, subtypes, and molecules.

##### Therapeutic Strategies and Clinical Applications

**Figure 2:**
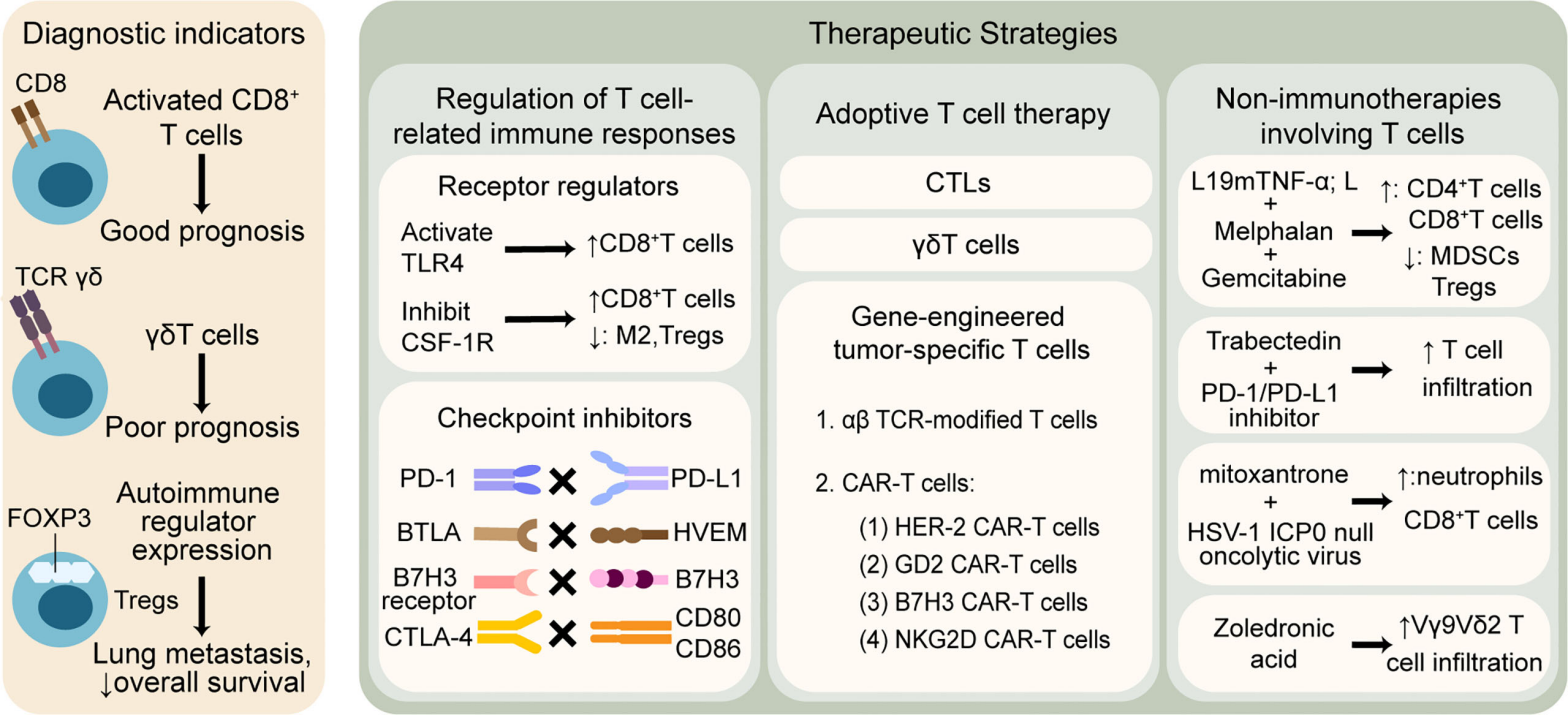
T cell-related therapeutic strategies and clinical applications in osteosarcoma. T cells are widely explored to predict outcomes and promote anti-tumor treatment. The left column shows that T cell infiltration profile may be associated with different prognosis. The right column illustrates that therapies involving T cells are mainly divided into three categories: 1) regulation of T cell-related immune responses by modulating receptors and blocking checkpoints, 2) adoptive T cell therapy based on CTLs, γδT cells and gene-engineered tumor-specific T cells, 3) non-immunotherapies containing T cell-related mechanisms. TLR, toll-like receptor; CSF-1R, colony-stimulating factor 1 receptor; M2, tumor associated macrophages M2 phenotype; PD-1, programmed cell death protein-1; PD-L1, programmed cell death protein ligand-1; BTLA, B And T-lymphocyte attenuator; HVEM, herpesvirus entry mediator; CTLA-4, cytotoxic T-lymphocyte-associated protein-4; CTLs, cytotoxic T lymphocytes; TCR, T cell receptor; CAR-T cells, chimeric antigen receptor T cells; HER-2, human epidermal growth factor receptor-2; GD2, disialoganglioside; NKG2D, natural killer group 2 member D; TNF-α, tumor necrosis factor-alpha; MDSCs, myeloid-derived suppressor cells; HSV, herpes simplex virus.

Given that T cells play a significant role in the immune microenvironment of osteosarcoma, T cell-related applications show vigorous vitality, the mechanisms of which can mainly be divided into the following aspects: 1) T cell infiltration profile used as an auxiliary indicator of diagnosis, such as disease staging, patient clustering, prediction of metastasis, drug resistance, and survival outcomes; 2) immunotherapies targeting T cell-related immune responses, including strengthening the function of effector T cells and weakening the inhibitory effect of Tregs; 3) adoptive T cell therapy, including CTL, γδT, and gene-engineered tumor-specific T cells; and 4) non-immunotherapies involving T cell-related pathways. All four aspects are discussed in order below, which is illustrated in **Figure 2**.

First, T cells are potential prognostic predictors and assistant indicators for clinical diagnosis. Weak immunosuppressive signals and strong T-cell immune responses have been found to be significantly associated with improved outcomes in osteosarcoma patients (57). The number of activated CD8^+^ T cells in tumor lesions of osteosarcoma patients has been found to be higher in men than in women (80). A study claimed that CD8^+^ T cells might be associated with a good prognosis, whereas γδT cells have a poor prognosis (58). Autoimmune regulator expression is an indispensable transcription factor for T cells, resulting in peripheral immune tolerance, which develops and obtains central tolerance in the thymus. Matsuda et al. (81) analyzed 43 biopsy samples of conventional osteosarcoma and found that autoimmune regulator expression was expressed in 58.1% of the samples and was related to increased Tregs (FOXP3^+^), lung metastasis, and reduced overall survival. The results suggest that autoimmune regulator expression might be an ideal prognostic indicator and a satisfying target for drug design. Based on the above studies, it is not difficult to conclude that if the infiltration of T cells in the surgically removed tumor biopsy can be carefully analyzed, it may provide guidance for the next step of treatment and predict the prognosis.

Second, T cell and T cell-induced immune responses are common targets in immunotherapies. Toll-like receptor is a key molecule involved in innate immunity, and acts as a bridge between nonspecific and specific immunity. Yahiro et al. (82) found that activation of the Toll-like receptor 4 signaling pathway could further stimulate CD8^+^ T cells in murine models, thereby inhibiting osteosarcoma progression. Fujiwara et al. (30) reported that the colony-stimulating factor 1 receptor inhibitor PLX3397 could consume TAMs and Tregs (FOXP3^+^) and enhance CD8^+^ T cell infiltration in primary and metastatic lesions. This phenomenon has been observed both *in vitro* and *in vivo*. *In vitro*, PLX3397 inhibited colony-stimulating factor-1 or tumor-conditioned media stimulation of pERK1/2 and reduced the pro-tumor M2 polarization of TAMs. In an osteosarcoma orthotopic xenograft model, systemic administration of PLX3397 significantly inhibited primary tumor growth and lung metastasis, contributing to improved metastasis-free survival. Currently, popular immune checkpoint inhibitors are being explored in the field of osteosarcoma treatment. Yoshida et al. (83) validated that anti-PD-1 antibody (4H2) could decrease Treg infiltration in subcutaneously implanted models of murine osteosarcoma cell line LM8, and finally, tumor volume decreased in size and overall survival was prolonged. Cascio et al. (84) reported that PD-1/PD-L1 was not the only immune checkpoint axis in human osteosarcoma lesions; herpesvirus entry mediator (HVEM/CD270) and indeterminate receptor to B7H3/CD276 were also expressed. The expression of these three immune checkpoints was significantly higher in metastatic lesions than in the primary lesions. The levels of the three ligands were positively correlated with each other and with peritumoral T-cell infiltration. Therefore, owing to low intratumor effector T cell infiltration in osteosarcoma, combined therapies of immune checkpoint inhibitors may be used to magnify immune infiltration, improve the immune microenvironment, and finally repress tumors in the future. Some researchers have combined immune checkpoint inhibitors with other therapies to further optimize osteosarcoma treatment. He et al. (85) observed that PD-1/PD-L1 therapy combined with L-arginine improved the therapeutic effect in immunocompetent BALB/c mouse models. L-arginine significantly increased the infiltration of CD8^+^ T cells, splenic CD8^+^ T cells, serum IFN-γ, and anti-α-PD-L1 antibody to prevent the exhaustion of CD8^+^ T cells and promote the expression of IFN-γ, granzyme B, and perforin. The combination of L-arginine and PD-1/PD-L1 immunotherapy significantly increased overall survival in mice; therefore, the addition of L-arginine may be a future direction. Takahashi et al. (86) found that a combination of dual checkpoint blockade therapy (anti-PD-L1 and anti-cytotoxic T-lymphocyte-associated protein-4) and X-ray irradiation could control primary osteosarcoma and diminish metastasis *in vivo*, which was associated with increased recruitment of CD8^+^ T cells and decreased infiltration of Tregs. Immune checkpoint inhibitors have not been shown to be significantly effective in all the studies. Nasarre et al. (87) investigated the role of a monoclonal antibody targeting humanized secreted frizzled-related protein 2, a protein that promotes angiogenesis and metastasis, in metastatic osteosarcoma resistance to PD-1/PD-L1 inhibitors, and its impact on T cells. They found that humanized secreted frizzled-related protein 2 monoclonal antibody inhibited T cell proliferation and osteosarcoma metastasis by lowering the expression of NFATc3, CD38, and PD-1.

Third, adoptive T-cell therapy is popular and is flourishing in osteosarcoma. Adoptive T cell transfer is one of the current research hotspots, which involves the introduction of specific T cells amplified *in vitro* into patients to supplement and enhance T cell-related immunity. Common applicable T cells include CTL, γδT, and gene-engineered tumor-specific T cells (88). In particular, gene engineering is moving to the center stage of osteosarcoma. Osteosarcoma-associated antigens are numerous and complicated, mainly represented by activated leukocyte cell adhesion molecules (CD166), B7H3, and epidermal growth factor receptor. T cells can be loaded with specific osteosarcoma-associated antigens by gene engineering, among which αβT cell receptor-modified T cells, chimeric antigen receptor T cells (CAR-T cells), especially HER-2 CAR-T cells, disialoganglioside GD2 CAR-T cells, and B7H3 CAR-T cells have been discussed the most (89). CAR-T cell therapy has progressed to the third generation with preliminary advances in hematologic tumors and neuroblastoma, but is still in the exploratory stage in osteosarcoma (90). Fernandez et al. (91) verified the safety and effectiveness of installing natural killer group 2 member D (NKG2D) CARs containing 4-1BB and CD3z signaling domains in CD45RA^−^ T cells through lentiviral transduction *in vivo* and *in vitro*. They found that the anti-tumor activity of NKG2D-CAR memory T cells was enhanced by strengthening the interactions between NKG2D ligands and receptors in osteosarcoma. However, it is not enough to increase the adaptation of effector T cells to osteosarcoma; it is also crucial to facilitate their proliferation, prolong their lifespan, enhance their resistance to the inhibitory immune microenvironment, and promote their susceptibility to tumor cells. Gene-engineered T cells can be used in treatment in the future, with more precise targets, flexible controllability, and richer functions.

Fourth, many non-immunotherapies are relevant to the regulation of immune microenvironmental pathways. Mortara et al. (92) found a significant increase in CD4^+^ and CD8^+^ T cells and a decrease in MDSCs and Tregs in the microenvironment of osteosarcoma syngeneic mouse tumor models, which responded well to the combination of targeting angiogenesis L19 tumor necrosis factor alpha (L19mTNF-α; L), melphalan, and gemcitabine. Atti et al. (93) showed increased T cell infiltration in the microenvironment of an osteosarcoma murine model treated with the alkylating agent trabectedin. However, CD8^+^ T cells were exhausted in no time, which may be due to the high expression of PD-1. Therefore, the team suggested the addition of PD-1/PD-L1 blockers to compensate for this failure to achieve better anti-tumor effects. Belisario et al. (94) found that the ratio of ATP-binding cassette transporter (ABC)-A1, an activator of anti-tumor Vγ9Vδ2 T cells, to ABC-B1, an inducer of chemotherapy resistance, could indicate chemo-immune resistance. In addition, zoledronic acid increased the sensitivity of drug-resistant osteosarcoma cells by enhancing intratumor apoptosis and the ratio of ABC-A1 to ABC-B1 and Vγ9Vδ2 T-cell infiltration. Workenhe et al. (95) reported that the combination of HSV-1 ICP0 null oncolytic virus KM100 and mitoxantrone, an immunogenic cell death-inducing chemotherapeutic drug, could significantly increase the survival benefit by increasing the infiltration of CD8^+^ T cells and neutrophils in the osteosarcoma microenvironment of BALB/c mice bearing HER-2/neu TUBO-derived tumors. The oncolytic HSV-1 did not reverse tumor immune tolerance *in vitro*, indicating that the two drugs might share some overlap in pharmacological mechanisms to achieve the effect that one plus one was greater than two.

Furthermore, some T cell-associated therapies have not yet achieved initial success, but they can inspire researchers. The addition of immunomodulatory cytokines such as IL2, IL15, and liposomal-muramyl tripeptide phosphatidylethanolamine might induce T cell proliferation and differentiation to improve the survival outcomes of osteosarcoma patients, but the evidence is insufficient. Studies on specific monoclonal antibodies and bispecific antibodies targeting osteosarcoma cells are also ongoing, but there is abundant evidence. Anti-tumor vaccines are thought to be able to clear small residual lesions in the body by inducing active or passive specific immunity, and several clinical trials of sarcoma are underway (**Table 1**), which is expected in the field of osteosarcoma (90). In summary, the function of T cells in the immune microenvironment of osteosarcoma and their interactions with other components have not been fully recognized, and relevant therapies remain in the preliminary stage of exploration. Further research is required to expand this field and inspire applications.

**Table 1:** Clinical trials of anti-tumor vaccines in sarcoma.

NCT number	Phases	Diseases	Vaccines
NCT00923351	I, II	Ewing’s sarcoma, undifferentiated/embryonal sarcoma, desmoplastic small round cell tumor, synovial cell sarcoma, rhabdomyosarcoma	Tumor purged/CD25 depleted lymphocytes with tumor lysate/KLH pulsed dendritic cell vaccine
NCT00405327	II	Sarcoma, neuroblastoma, Wilm’s tumor	Tumor lysate-pulsed dendritic cell vaccine
NCT00001566	II	Ewing’s sarcoma, rhabdomyosarcoma	Autologous dendritic cell vaccine
NCT01141491	II	Sarcoma	Trivalent ganglioside vaccine
NCT01241162	I	Ewing’s sarcoma, osteogenic sarcoma, rhabdomyo sarcoma, synovial sarcoma, neuroblastoma	Autologous dendritic cell vaccine
NCT00069940	I	Sarcoma, central nervous system tumors, gastrointestinal stromal tumor	Telomerase 540-548 peptide vaccine
NCT00003023	I	Sarcoma, neuroblastoma	BCG vaccine, monoclonal antibody A1G4 anti-idiotype vaccine
NCT00199849	I	Sarcoma, prostate cancer, bladder cancer, non-small cell lung cancer, esophageal cancer	NY-ESO-1 plasmid DNA cancer vaccine

#### 2.2.2 B Cells and Antibodies

B cells are derived from lymphoid stem cells in the bone marrow and reside in the lymphoid follicles of the peripheral lymphoid organs when they mature. B cells are not only the protagonists of humoral immunity by producing antibodies but are also a type of antigen-presenting cells involved in immunoregulation. According to the activation stage, B cells can be divided into three categories: initial B cells, memory B cells, and effector B cells/plasma cells, among which the latter is the main source of antibodies. Regulatory B cells are a type of B cells with immunosuppressive effects. Regulatory B cells inhibit CD4^+^ T cells, CTLs, macrophages, and DCs by secreting inhibitory cytokines such as IL10, TGF-β, IL35, and expressing membrane surface regulatory molecules such as FasL and CD1d, and promote the transformation of T cells into Tregs, thus weakening anti-tumor immune responses (96). Although humoral immunity is not the predominant mechanism of the anti-tumor immune responses, it plays an indispensable role. Overall, B cells and humoral immunity are receiving increasing attention in anti-tumor immunity, and breakthroughs are expected in this area.

##### Therapeutic Strategies and Clinical Applications

The infiltration of B cells in osteosarcoma is complex, with differences in cell subtypes and patient sex. Yang et al. (80) obtained data on osteosarcoma cases from The Cancer Genome Atlas and performed a comprehensive assessment of the infiltration of immune cells. They found more memory B cells and activated B cells in osteosarcoma lesions in men than in women. Li et al. (97) analyzed the immune cells in the microenvironment of osteosarcoma, Ewing’s sarcoma, multiple myeloma, and cancer bone metastases and found that osteosarcoma patients with high infiltration of B cells had a better prognosis and activated B cells were positively correlated with survival.

Therefore, infiltration of effector B cells may be a good prognostic indicator. Research on B cells in osteosarcoma is still very limited, and new therapies based on B cells lack satisfactory results.

Antibodies produced by B cells and plasma cells proliferate and differentiate from memory B cells and mainly exist in serum, tissue fluids, secretory fluids, and on the surface of certain cells.

Antibodies regulate tumor growth and metastasis through antibody-dependent cell-mediated cytotoxicity, modulatory effects, activation of complement, closure of tumor cell receptors, and alteration of tumor cell adhesion. Contrary to common sense, some antibodies can bind to antigens on the surface of tumor cells and block killing (98–100). Immunoglobulins are globulins with antibody activity or similar chemical structure domain antibodies that are mainly distributed in the serum or on B-cell membranes. Studies on antibodies and immunoglobulins in osteosarcoma are relatively few and need to be further investigated. IgE has been found to be associated with osteosarcoma development. Zhang et al. (101) conducted bioinformatic analysis on 19 osteosarcoma cases and six normal samples obtained from the Gene Expression Omnibus database and compared the differentially expressed genes, differentially methylated regions, copy number, and functional enrichment of the two groups. The results showed that hypermethylation in the fragment crystallizable region of immunoglobulin (Ig) E, high-affinity I, receptor for γ polypeptide was significantly associated with osteosarcoma development. IgE elevation is often seen in allergic diseases; therefore, the specific role of IgE epigenetic alterations in osteosarcoma immunity needs to be further explored.

##### Therapeutic Strategies and Clinical Applications

Immunoglobulins, their receptors, and their transporters can be used as predictors and diagnostic factors. Wang et al. (102) found that positive expression of polymeric immunoglobulin receptor, the transporter of dimeric IgA, and pentameric IgM, was significantly associated with poor prognosis in patients with osteosarcoma, indicating that polymeric immunoglobulin receptor might be a good prognostic biomarker. Guerra et al. (103) analyzed biochemical and immunological parameters in the saliva of healthy children and children with cancer before and after antineoplastic treatment, including osteosarcoma. The total concentration of IgA in the saliva of children with cancer was significantly lower than that in healthy children, independent of antineoplastic treatment. This noninvasive test provides a new clue for the diagnosis and treatment of childhood cancer. In addition, antibodies specific for osteosarcoma-associated antigens are the mainstay of humoral immunity and the raw material for the design of anti-tumor drugs. Receptor tyrosine kinase-like orphan receptor 2 has been found to be a highly expressed osteosarcoma-associated antigen, and its antibodies are potential drugs. Hellmann et al. (104) fabricated a clinically used monoclonal antibody with high affinity to receptor tyrosine kinase-like orphan receptor 2 using human Ig transgenic animals, providing a new idea for osteosarcoma immunotherapy. Autoantibodies can also be used for etiological studies. Mazzoni et al. (105) discovered that IgG reacting with Simian virus 40 mimotopes was significantly higher in the sera of osteosarcoma patients than in those of breast cancer patients, undifferentiated nasopharyngeal carcinoma patients, and healthy people, demonstrating an association between Simian virus 40 and osteosarcoma pathogenesis. The Ig superfamily may conceal the secrets to osteosarcoma progression and metastasis. Leukocyte-associated immunoglobulin-like receptor-1 is a collagen receptor of the Ig superfamily, and the related pathways in lymphocytes and monocytes have received increasing attention. Leukocyte-associated immunoglobulin-like receptor-1 overexpression decreased Glut1 and epithelial-mesenchymal transition-related molecules, thereby inhibiting osteosarcoma cell metabolism and metastasis and providing a new target for slowing osteosarcoma progression (106). Although studies on antibodies and immunoglobulins in osteosarcoma are scarce, they may exert considerable influence on the diagnosis, treatment, and scientific research of osteosarcoma in the future.

#### 2.2.3 Natural Killer Cells

NK cells are a class of innate lymphoid cells that express the intracellular transcription factors E4BP4^+^ and CD3^-^CD19^-^CD56^+^CD16^+^ on their surface. NK cells are widely distributed in the blood, peripheral lymphoid tissues, liver, and spleen. NK cells have been found to not only kill tumor cells directly but also control tumor progression and metastasis. NK cells express a variety of cytokine receptors related to chemotaxis and activation and can be recruited to the tumor microenvironment to kill tumor cells by releasing perforin, granzyme, and tumor necrosis factor-α and expressing FasL (107). The PD-1/PD-L1 axis can regulate the anti-tumor effects of NK cells. Zhang et al. (108) found that blocking the PD-1/PD-L1 axis with a PD-L1 antibody, which inhibited NK cell toxicity by secreting granzyme B, could enhance the killing effect of NK cells on human osteosarcoma cells. Yang et al. (80) conducted a comprehensive analysis of immune infiltration in the osteosarcoma microenvironment and concluded that male patients had more NK cells than did female patients. Lazarova et al. (109) discovered that NK cells were suppressed, but TGF-β expression increased in the osteosarcoma microenvironment. The mechanisms explained were that TGF-β could promote angiogenesis, bone remodeling, and cell migration by inhibiting the expression of activation receptor NKG2D and reducing the release of killing perforin of NK cells. To overcome these negative effects and induce resistance of NK cells to TGF-β, Kisseberth et al. (110) continuously exposed NK cells to low-dose TGF-β and IL2 *in vitro*, thereby alleviating the degree of immunosuppression in the canine osteosarcoma microenvironment, which might be applied to the treatment of human NK cells in the future.

##### Therapeutic Strategies

For cancer cells evading adaptive immune surveillance by antigen shedding, lowering major histocompatibility complex-I, and inhibiting T cells, NK cells alone or in combination show great application prospects. Attempts have been focused in three directions: adoptive NK cells, cytokine-based targeting therapies that enhance the immune activity of NK cells, and chimeric antigen receptor-NK cells (CAR-NK). Adoptive NK cells have achieved initial success in the treatment of osteosarcoma. The basic principle is to compensate for the hypothetically existing immune deficiency and reactivate the suppressed NK anti-tumor immunity. NK cells used for treatment can be obtained from sources such as autologous or alien peripheral blood, umbilical cord blood, hematopoietic progenitors, and pluripotent stem cells. The advantage is that it is safe, and there is no graft vs. host disease, such as CAR-T cells and immune checkpoint inhibitors (111). Mismatched allogeneic donors may have a greater anti-tumor effect than matched or autologous NK cells in osteosarcoma (112). Chu et al. (113) combined N-803 (IL15 super agonist) and dinutuximab (monoclonal antibody targeting disialoganglioside GD2) to treat *ex vivo* expanded peripheral blood NK cells. They found that the toxicity of expanded peripheral blood NK cells was enhanced, and this effect was verified *in vivo*, with significantly longer survival in osteosarcoma mice. In addition, Buddingh et al. (114) found that utilizing IL15 to treat allogeneic and autologous NK cells could restore the sensitivity of chemotherapy-resistant osteosarcoma through DNAX accessory molecule-1 and NKG2D pathways. These two studies emphasized the application value of IL15, an NK cell-activating agent, in osteosarcoma. CAR-NK is a novel and promising therapy for loading NK cells with specific antibodies, which has been observed to have therapeutic effects in Ewing’s sarcoma and B-cell leukemia (115). However, there is still a lack of research on CAR-NK cells in osteosarcoma. Research on NK cell biology, checkpoint inhibition, CAR technology, and the expansion of autologous and allogeneic NK cells is of great value and urgently needed.

### 2.3 Non-Immune Cells

#### 2.3.1 Mesenchymal Stem Cells

CSCs are competitive clones that drive tumorigenesis. CSCs are considered the key reason for the huge heterogeneity of osteosarcoma and cause recurrence, metastasis, and drug resistance (116). Xu et al. (117) divided osteosarcoma patients into two clusters based on osteosarcoma tumor stem cell-related genes. Cluster 1 had a higher immune infiltration score and a better prognosis than Cluster 2. The Cluster 1 immune microenvironment is characterized by fewer follicular helper T cells, M0 macrophages, and more CD8^+^ T cells. These results suggest that CSCs are potentially associated with the immune microenvironment landscape in osteosarcoma, which means that different CSCs may develop different immune infiltrates and have different prognoses. There is a lot of research indicating that osteosarcoma originates from MSCs. MSCs are adult multipotent stem cells distributed in various tissues of the body, especially in the bone, adipose tissue, and dental pulp.

MSCs play an important role in osteosarcoma tumorigenesis by regulating immune responses and by inducing cell fusion and differentiation (118). A study on osteosarcoma histogenesis suggested that naive MSCs and tumor-derived MSCs may exert different effects on osteosarcoma development. Naive MSCs were found to have both anti-tumor and pro-tumor effects. Tumor-derived MSCs have been shown to promote tumor cell proliferation, increase CSCs proportion, facilitate epithelial-mesenchymal transition, and exhibit strong immunosuppressive activity (119). There are two non-immune mechanisms by which MSCs promote osteosarcoma cell proliferation and metastasis: first, the interaction between osteosarcoma cells and MSCs involving IL8 and aquaporin 1; second, abnormal gene expression, such as *Rb*, *C-MYC*, *TP53*, *K-Ras*, and *IHH* promote the transformation of MSCs into osteosarcoma cells (120–125). MSCs have also been found to transform into cancer-associated fibroblasts when exposed to osteosarcoma cells, which could significantly accelerate the proliferation, migration, and invasion of osteosarcoma cells. This process involves monocyte chemotactic protein 1, growth-related oncogene-alpha, TGF-β, and intercellular adhesion molecule 1. Cancer-associated fibroblasts can also secrete extracellular matrix responsible for intercellular communication, cell adhesion, and proliferation to shield malignant phenotypes and enrich tumor heterogeneity. Moreover, the effects of MSCs and osteosarcoma cells were similar. Under MSC stimulation, osteosarcoma cells can induce endothelial cell migration and invasion and promote angiogenesis (126–129). When it comes to immunity, MSCs could secrete anti-inflammatory substances and inhibit pro-inflammatory substances to help osteosarcoma cells escape immune surveillance, mediated by autocrine or paracrine extracellular vesicles (EVs), particularly exosomes (130). Lagerweij et al. (131) found that MSCs inhibit T cell proliferation and immune responses by secreting EVs containing miRNAs/RNA and proteins. Zhang et al. (132) reported that MSC-EV-associated TGF-β and IFN-γ can promote the transformation of mononuclear cells into Tregs. In addition to influencing T cell-associated immune responses, MSCs have been found to inhibit the immune effects of B cells. Khare et al. (133) found that MSC-EVs increased the levels of C-X-C motif chemokine ligand 8 and MVB1 RNA, which could reduce IgM in B cells. MSCs can also promote the M2 polarization of TAMs by secreting IL6 (134). Some cytokines, such as IL10, hepatocyte growth factor, leukemia inhibitory factor, C-C motif chemokine ligand 2, vascular endothelial growth factor-C, and C-C motif chemokine ligand 20 (135) are also very important in the increased migration of MSC-EVs to the tumor microenvironment and inflammation inhibition.

##### Therapeutic Strategies

The application of MSCs has focused on two aspects: 1) regulation of the signaling pathways and secretion behavior of MSCs and 2) delivery of drugs by MSCs. For example, Alvaro et al. (136) loaded MSCs with an oncolytic virus and granulocyte-colony stimulating factor to increase immune infiltration and alleviate tumor growth. However, it should be noted that the pharmacokinetic characteristics of MSCs vectors require extensive studies to ensure both efficacy and safety (118). However, a few studies have shown that MSCs can effectively inhibit osteosarcoma recurrence, proliferation, and metastasis. Aanstoos et al. (137) injected MSCs directly into the tumor mass of osteosarcoma mice and found that tumor expansion and local recurrence were controlled. In contrast, the intravenous administration of MSCs to osteosarcoma mice promoted lung metastasis. The researchers attributed the anti-tumor activity to the promotion of apoptosis, restraint of angiogenesis, and regulation of immune responses. The reason for this contradiction may lie in an unclear understanding of the different sources and functional characteristics of MSCs in the academic community. In the field of osteosarcoma, attempts to target pro-tumor MSCs subtypes or amplify anti-tumor MSCs subtypes are still in the exploration stage, and research on the development of MSC-associated exosomes or other EVs is still insufficient. It is hoped that a breakthrough will be realized in the near future.

#### 2.3.2 Circulating Tumor Cells

Osteosarcoma cells exist not only in the tumor mass, but also in circulation, and are called CTCs. CTCs can escape local treatment, such as surgical resection and radiotherapy and survive in small quantities under systemic treatment, which results in the metastasis and recurrence of osteosarcoma. Several studies have shown that CTCs play a specific role in the immune microenvironment of osteosarcoma. Zhang et al. (138) found that the inhibition of IL6 could suppress osteosarcoma cell proliferation and reduced CTCs. The phenomenon observed in *in vitro* experiments was that recommendation human interleukin 6 activated Janus-activated kinase/signal transducers and activators of transcription 3 and mitogen-activated protein kinase/extracellular signal regulated kinase1/2 pathways. Both pathways promote osteosarcoma cell proliferation, but only the former promotes cell migration. This effect was also confirmed in a nude mouse model of human osteosarcoma. Given that activated Janus-activated kinase/signal transducers and activators of transcription 3 can enhance immunosuppression, it is not difficult to speculate that CTCs are related to the formation of an immunosuppressive microenvironment in osteosarcoma.

In addition to IL6, IL8 has also been found to be a positive factor in the promotion of osteosarcoma progression by CTCs. The main biological activity of IL8 is to attract and activate neutrophils, eosinophils, basophils, and lymphocytes. Liu et al. (139) successfully isolated and cultivated self-seeded CTCs and discovered that IL8 could promote tumor growth and lung metastasis *in vitro* and *in vivo*. Therefore, inhibiting IL8 might exert a therapeutic effect, and a combination with activators or inhibitors of other cytokines would be encouraged. Although the mechanisms of the interaction between CTCs and tumor immunity are not clear, CTCs are potential predictive markers and drug targets. Elimination of CTCs is expected to be a symbol of a more thorough efficacy of anti-tumor therapies.

## 3 Complement System

The complement system is a protein reaction system consisting of more than 30 components with precise regulation and is widely present in the serum, tissue fluid, and cell membrane surface. In addition to innate immunity to pathogens, the complement system participates in the immune regulation of inflammation and tumors. The majority of current studies suggest that complement system activation has anti-tumor effects by killing tumor cells through complement-dependent cytotoxicity, whereas other studies suggest that complement system activation has pro-tumor potential in a special immune microenvironment.

### Clinical Applications

Chen et al. (140) found that the expression level of C1q (C1qA, C1qB, and C1qC) was positively correlated with the survival time of patients with osteosarcoma and negatively correlated with the percentage of surgical pathological necrosis, indicating that C1q is likely to be a positive prognostic factor. Gene set enrichment analysis showed that immune-related genes were significantly enriched in the group with high C1q expression, indicating that high expression of C1q implies strong immune function and better prognosis. CiberSort proportional analysis showed that the expression level of C1q was positively correlated with M1 and M2 macrophages and CD8^+^ T cells, which added to the specific mechanisms by which C1q interacts with various cells in the immune microenvironment. Similarly, another study published in 2021 suggested that C1q could predict osteosarcoma metastasis and prognosis. Huang et al. (141) found that C1qA, C1qB, and C1qC levels were positively correlated with the number of follicular helper T cells, CD8^+^ T cells, and memory B cells. Moreover, the expression of these three key genes was significantly lower in metastatic osteosarcoma cell lines than in non-metastatic osteosarcoma cell lines. This finding also indicates that C1q is a positive prognostic factor. However, Jeon et al. (142) found that after the addition of normal human serum, human bone osteosarcoma epithelial cells (U2-OS) activated the alternative pathway of the complement system, resulting in the generation of more vascular endothelial growth factor-A and fibroblast growth factor 1 and promotion of angiogenesis in models *in vitro*, regulated by the phospho-ERK signaling pathway. Moreover, the expression of negative complement regulatory proteins, such as CD46, CD55, and CD59 and endogenously expressed properdin or C3, was reduced in normal human serum-treated U2-OS cells. Despite the above research, the entire landscape of the complement system and its role in the immune microenvironment of osteosarcoma are still unknown, which may be an interesting direction for future research.

## 4 Exosomes

EVs are lipid-coated vesicles secreted by cells into the extracellular space, containing nucleic acids (DNA, RNA, and microRNAs), proteins, lipids (eicosanes, fatty acids, and cholesterol), and intact organelles. In osteosarcoma, exosomes are representative special heterogeneous vesicles with a diameter of 30–150 nm produced by direct outward budding of the cell membrane (143). Exosomes play an important role in the proliferation, metastasis, and drug resistance of osteosarcoma. Many reviews have elaborated on these mechanisms, and this review attempts to summarize the role and application of exosomes related to immunity.

Immune effects of exosomes have been observed in other tumors also. For example, in mesothelioma, TGF-β1 in exosomes significantly downregulates NKG2D on the surface of CD8^+^ T cells and NK cells, inhibits lymphocyte activation, and further impedes tumor cell recognition (144). Exosomes containing TGF-β1 in mesothelioma have also been found to inhibit IL2-mediated lymphocyte proliferation more strongly than does soluble TGF-β1, thereby increasing immune escape (145). In breast cancer, TGF-β in exosomes induces monocyte differentiation and accumulation of immature MDSCs (146). Exosomes produced by osteosarcoma cells have been reported to have a bidirectional effect on tumors; however, there are only a few studies on their immune effects. Exosomes have tumor-associated antigens on their surfaces, which can interact with antigen-presenting cells and induce tumor-specific toxic T-cell immune responses (75). Similar to exosomes secreted by osteoblasts, exosomes secreted by osteosarcoma cells also have immunosuppressive effects, even at a much higher degree. Exosomes containing TGF-β, α-fetoprotein, and heat shock proteins slow T cell proliferation, prevent effector T cells from working, promote a regulatory (FOXP3^+^) CD4^+^ phenotype, and lower the expression of the activation marker CD25 on CD8^+^ cells (147). Exosomes secreted by metastasized osteosarcoma cells have been found to induce M2 polarization *via* TGF-β2, thus promoting further invasion and metastasis (148). Exosomes can also disrupt the activity of NK cells and DCs *via* TIM-3 (149, 150).

### Therapeutic Strategies and Clinical Applications

The application of exosomes mainly includes two aspects: 1) exosomes as diagnostic and prognostic indicators, and 2) exosomes as drug carriers because of their good biocompatibility and low immunogenicity. Wang et al. (151) reported that the serum levels of exosomal PD-L1 and N-cadherin in patients with osteosarcoma could accurately predict metastasis. Shimbo et al. (152) encapsulated artificial synthetic therapeutic miRNA-143 in exosomes and released them into the osteosarcoma microenvironment, and found that the therapy significantly reduced osteosarcoma cell migration. Although exosomes are expected to be potential drug delivery vectors in cancer, packaging drugs and modifying exosomes creatively to obtain more controlled and stable release and fewer adverse effects is one of the directions for future research. **Table 2** lists clinical trials on exosomes in other malignant tumors, indicating that exosomes may also be versatile in osteosarcoma research.

**Table 2:** Clinical trials on exosomes in other malignant tumors.

NCT number	Diseases	Exosomes	Functions
NCT02702856	Prostate cancer	Urinary exosome gene signature	Diagnosis
NCT03830619	Lung cancer	Serum exosomal long noncoding RNAs	Diagnosis
NCT03032913	Pancreatic ductal adenocarcinoma	CTCs and onco-exosomes	Diagnosis
NCT04720599	Urologic cancer	ExoDx Prostate Intelliscore	Diagnosis
NCT02662621	Malignant solid tumors	HSP70-exosomes in the blood and urine	Diagnosis
NCT03911999	Prostate cancer	Urinary exosomal microRNA	Prediction
NCT03031418	Prostate cancer	ExoDx Prostate Intelliscore	Prediction
NCT03895216	Bone metastases	Deregulated miRNAs in the circulating exosomes	Prediction
NCT02862470	Thyroid cancer	Urinary exosomes	Prediction
NCT01159288	Non-small cell lung cancer	Vaccination with tumor antigen-loaded dendritic cell-derived exosomes	Treatment

## 5 Conclusions, Limitations, and Perspectives

Osteosarcoma is a primary malignant bone tumor that mainly occurs in children, adolescents, and the elderly, with a poor prognosis. The immune microenvironment of osteosarcoma is a complex system with high heterogeneity, which is closely related to the escape of tumor cells from immune surveillance, uncontrolled proliferation, and metastasis. Amplifying the effect of anti-tumor immunity and inhibiting pro-tumor immunity may be a promising approach to eliminate microscopic tumor lesions and CTCs. This review systematically summarizes the roles of cellular and non-cellular components in the immune microenvironment of osteosarcoma and the progress in related therapeutic strategies and clinical applications over the last 10 years, especially in the last 5 years, providing a reference for future research, diagnosis, and treatment of osteosarcoma.

However, the research above is far from sufficient for the following reasons. 1) First, the tumor immune microenvironment is variable, as reflected by variations in disease type and subtype, disease course, and individual. The current understanding of the roles of components of the immune microenvironment of osteosarcoma is still at the stage of borrowing analogous findings from other solid tumor types, which may be vague and one-sided. 2) Second, most studies remain in the preclinical stage, and evidence of translational medicine is insufficient. 3) Finally, the immune system influences the entire body, and immune-related therapies should be both safe and effective. If not employed properly, they can lead to new and serious diseases; therefore, adverse effects should be considered seriously.

Based on this, some future directions are proposed: 1) Diagnosis: Explore the multi-factor predictive model, diagnosis model, and grading scores of osteosarcoma that contain immune components to promote precision treatment. 2) Treatment: Develop new approaches that are more effective than existing treatment schemes under the premise of safety, try to combine immunotherapy with other therapies, and pursue cheaper and faster manufacturing. 3) Research: Find more ideal identification markers of immune cells and non-immune cells, determine interactions among immune microenvironment components, find more ideal therapeutic targets, and combine multidisciplinary knowledge and multi-technical assistance for research.

## Author Contributions

TZ and JH contributed to conception, designed of the outline, and wrote the manuscript. LY and ZC helped to search for and systematically sort out the literature and material. LY, WS, YH, and JX polished up the writing. WS, YH, and JX conducted oversight and took leadership responsibility for the whole research planning and execution. All authors contributed to manuscript revision, read, and approved the submitted version.

## Funding

This work was supported by National Natural Science Foundation of China (grant number 81872177; 81972517) and Shanghai Rising-Star Program (grant number 20QA1408000).

## Conflict of Interest

The authors declare that the research was conducted in the absence of any commercial or financial relationships that could be construed as a potential conflict of interest.

The handling editor declared a past co-authorship with the authors LY and YH.

## Publisher’s Note

All claims expressed in this article are solely those of the authors and do not necessarily represent those of their affiliated organizations, or those of the publisher, the editors and the reviewers. Any product that may be evaluated in this article, or claim that may be made by its manufacturer, is not guaranteed or endorsed by the publisher.

## Abbreviations

ABC, ATP-binding cassette transporter; CAR-NK, chimeric antigen receptor-NK cells; CAR-T cells, chimeric antigen receptor T cells; CSC, cancer stem cell; CTCs, circulating tumor cells; CTLs, cytotoxic T lymphocytes; DCs, dendritic cells; EVs, extracellular vesicles; G-MDSCs, granulocytic MDSCs; IFN-γ, interferon-gamma; Ig, immunoglobulin; IL, interleukin; MCs, mast cells; MDSCs, myeloid-derived suppressor cells; M-MDSCs, monocytic MDSCs; MSCs, mesenchymal stem cells; NK cells, natural killer cells; NKG2D, natural killer group 2 member D; PD-1, programmed cell death protein-1; PMN-MDSCs, polymorphonuclear MDSCs; STAT, signal transducer and activator of transcription; TAMs, tumor-associated macrophages; TANs, tumor-associated neutrophils; TGF-β, transforming growth factor-β; Th, helper T cells; TIM-3, T cell immunoglobulin and mucin domain-containing protein-3; Tregs, regulatory T cells.
